# “Don’t Just Do Something … Stand There!” Emergency Responders’ Peri-Incident Perceptions of Animal Owners in Bushfire

**DOI:** 10.3389/fvets.2017.00034

**Published:** 2017-03-16

**Authors:** Rachel Anne Nosworthy Westcott, Kevin Ronan, Hilary Bambrick, Melanie Taylor

**Affiliations:** ^1^Centre for Health Research, School of Medicine, Western Sydney University, Sydney, NSW, Australia; ^2^Bushfire and Natural Hazards Cooperative Research Centre, Melbourne, VIC, Australia; ^3^Professorial Research Fellow, Clinical Psychology, School of Health, Medical and Applied Sciences, Central Queensland University, Rockhampton, QLD, Australia; ^4^Head of School, Public Health and Social Work, Faculty of Health, Queensland University of Technology, Brisbane, QLD, Australia; ^5^Senior Lecturer Organizational Psychology, Macquarie University, Sydney, NSW, Australia

**Keywords:** bushfire, wildfire, animals, disaster, emergency, emergency responder, animal owners

## Abstract

**Introduction:**

Narrowing the awareness–preparedness gap in bushfires (wildfires) means that new strategies and tactics will be needed to improve human safety and survival in this increasingly frequent and severe globally significant natural hazard. One way to do this is to explore the peri-event experiences of novel demographic groups living and working in at-risk areas to determine how best to strengthen a collaborative, mutually beneficial interface with emergency responders. Thus, this study included participants from one novel demographic, animal owners, in combination with emergency responders. Animal owners themselves are a large, diverse group whose preparedness and response behavior has not been assessed with respect to their potential contribution to contemporary natural hazard management.

**Method:**

Data were collected using semi-structured interviews and focus group discussions from four emergency responder classifications who were asked about their perceptions of animal owners in bushfire. Thematic analysis was used for data analysis because of its flexibility and suitability to this pragmatic qualitative study. Results from the first of 10 themes, chosen for its “overview” properties, are discussed in this paper, and indicate that exploring the animal owner—emergency responder interface has the potential to generate useful additions to public policy and expansion of social theory.

**Conclusion:**

Analysis of these data in this paper supports the potential for positive outcomes gained by reciprocal collaboration between animal owners and emergency responders. Some simple practical solutions are evident and two major outcome streams are identified. These are (1) policy development and implementation and (2) etiology of decision-making. Considerations and recommendations for research examining the efficacy of these streams and solutions are provided.

## Introduction

The quotation in the title of this paper means that, to act effectively, it is first necessary to stop and dynamically risk assess a situation, and, even more significantly, to be in a position of confidence with a well-constructed, pre-prepared, and well-practised plan of action. Research shows that such a response can save, not waste, time, and may help reduce the rash, adrenalin-fuelled actions that can end in fatality (1, 2). This concept is counter-intuitively expressed by some responders as *hurry up and wait!* Being ready is the fulcrum about which effective bushfire response choices are made. Considered, timely and safe action—including coping appraisal and adaptive responses—both outside the fire season and within when threat is imminent, will usually promote the least noxious outcome. In this instance, the double negative is chosen deliberately because it is not the same as the “best” outcome.

This study records, documents, and analyses some of the experiences, expectations, and needs of communities who have “lived through” bushfire emergencies, and *expect to face this hazard again*; the ultimate aim of the study is to protect human life by making response behaviors safer and improving fire readiness and response routines. This paper examines the experiences and interactions of firefighters, police, and rescue officers of the State Emergency Service (SES) with animal owners in bushfire hazards, from the emergency responders’ perspective. The exploration of this interface aims to inform a collaborative path forward to strengthen shared responsibility, self-sufficiency, and reciprocal understanding to build trust and promote community engagement in future scenarios. A corollary purpose is to evaluate patterns of collaboration that might be generalized across other demographic groups within a community.

A case study of a bushfire at-risk regional center in South Australia—“the driest state in the driest continent” (3) was chosen as the research site because of its recent, and severe, fire history, and its diversity of animal ownership (4, 5). A pragmatic approach within a critical realist ontology and contextualist, experiential epistemology guided the research design due to the need to arrive at practical answers to issues of policy and practice (6–8).

The aim of this study’s overall data corpus, of which this data set is a part, is to explore an expansion of Protection Motivation Theory, to better theorize and understand the behavior of animal owners in bushfire situations (9). In part, this study was designed to develop new, meaningful preparedness initiatives to inspire and motivate the translation of knowledge into effective, adaptive action by all residents, and in particular, animal owners, of bushfire at-risk communities.

To date, the majority of academic literature about animal owners in emergencies is skewed toward the retrospective experiences only of pet owners (10). While such a focus may be a useful starting point, it is subject to recall problems and focuses on the views of only one set of animal owners. It also excludes emergency responders’ perceptions and in-field observations of animal owners’ behavior and reactions during an incident involving many species of animals, owned in a variety of contexts. Consequently, these experiences have not been investigated to identify new information that may be able to fill current gaps in contemporary emergency communication and warnings. This paper’s data set, therefore, asks the research question, *how can bushfire emergency responders’ experiences with animal owners help improve owner safety and survival?* It explores how emergency responders perceive animal owners (of any species and any number of animals) in the context of bushfire: their assessment of *what* owners do, and *how* they do it, with the goal of discovering *why* owners adopt a certain course of adaptive or maladaptive action. From this, adaptive behaviors can be confirmed and described. Importantly, maladaptive behaviors can be similarly identified, and (i) responses developed to either rectify or neutralize the actions and (ii) favored adaptive behaviors that enhance safety and survival may be usefully translated or applied.

Despite the provision of sophisticated, well publicized and widely accessible public education by fire authorities in Australia, messages of mitigation and readiness remain inconsistently received in the wider community across all hazards. Although *awareness* of the danger posed by bushfires seems to be increasing, the *awareness*–*preparedness gap* in community and individual residents’ survival plans is narrowing disproportionately slowly compared to the magnitude of resources applied to rectify this trend (11, 12). To help address this and to keep ahead of a climate change induced, worsening global fire threat, new strategies, and tactics, which resonate broadly with people—especially those in at-risk areas and demographics—need to be identified and implemented.

Fire can become an emergency when people, property, the environment, and other assets are impacted: the animal-owning public is challenged to properly and safely manage their animals, in addition to themselves, in emergency fire situations. Australia, like many Western countries, is a nation of animal lovers and animal owners. Sixty-three percent of Australian households own a companion animal (13), though the number owned by primary producers in rural and regional areas is much larger (14). Animal welfare is important, but should not be viewed in isolation, because it is frequently inextricably linked to human physical and, arguably more importantly, psychological health. Animals have a role as diffusers of social awkwardness, or as the means by which new relationships and introductions might form. They often change how people behave from day to day in the “routine” world: bringing solace, joy, achievement, profit, and sometimes sadness (15, 16). When faced with an emergency such as fire or flood, the presence of animals adds varying degrees of complexity to owners’ preparedness and planning. Yet, the needs of animal owners have not been specifically examined in the context of bushfire, despite the growing understanding of the strong link between effective animal management in an emergency and the saving of human life (17, 18).

As the title of this paper conveys, to act effectively in an emergency it is necessary to be in a position of confidence with a well-constructed, pre-prepared, and well-practiced plan of action. Close to 2,300 years ago, Aristotle wrote: *we are what we repeatedly do. Excellence, then, is not an act, but a habit*. In the context of this study, for “excellence” read, “preparedness,” which is a central organizing concept and an *a priori* major theme underpinning analysis of data in the current study. To “do” preparedness effectively requires its promotion from being regarded as an onerous task to a “business as usual” status—as routine as buying the groceries. Practicing readiness and bushfire preparedness frequently enough may lead, as Aristotle suggested, to safer behavior becoming instinctual. The basic human urge to save a dependent other at the expense of personal safety may never be overcome, but checks and balances, coping appraisal, and adaptive response—to “not just do something”—could mean that more can be achieved with less trauma and anxiety. Equally, the urge to prepare more for others’ (particularly dependents) needs compared to one’s own benefit is a leveraging point that can be used to motivate preparedness and doing it more effectively (19).

The Thematic Analysis (TA) below (7, 20) combines results and discussion for the first theme within this data set (Table 1). This approach is descriptive and interpretative and actively fluctuates between a more essentialist and a more constructionist analysis as the analytic story develops (6, 7).

**Table 1:** **Themes of the data corpus**.

Bushfire in kingdom animalia *(the taxonomic subject of this research)*		Paper, scissors, bushfire, action! *(threat appraisal, coping appraisal)*	
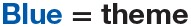	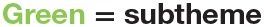		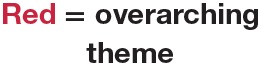
1. Bushfire and animals shouldn’t mix 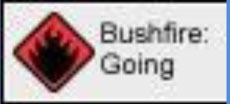 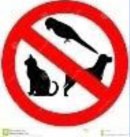	1a. *(Nearly) every animal has an owner (people)*	6. The purpose of life is a life of purpose *(responders)*	
	1b. On the sheep’s back *(farmers)*	7. A problem understood is a problem half solved *(adaptive response)*	
2. Be fire-fit: weekly is worth it! *(readiness)*		8. Ignorance is not bliss *(maladaptive and unsafe response)*	
3. Complexity of the social microclimate *(the 21st century family and their animals)*		9. Give me a home among the gum trees *(the tree-changers)*	9a. Effortless affordable luxury *(consumerism)*
	4. Trust		
	5. Information gathering		
10. When the dust settles *(recovery)*			

*The main themes listed in this table are (1) Bushfire and animals shouldn’t mix (2) be fire fit: weekly is worth it! (readiness) (3) complexity of the social microclimate (the 21st century family and their animals) (4) trust (5) information gathering (6) the purpose of life is a life of purpose (responders) (7) a problem understood is a problem half solved (adaptive response) (8) ignorance is not bliss (maladaptive and unsafe response) (9) give me a home among the gum trees (the tree-changers) (10) when the dust settles (recovery)*.

## Materials and Methods

### Positionality Statement: Ontology, Epistemology, and Methodological Approach to the Current Study

This paper reports on a data set comprising six interviews and three focus groups (*n* = 55) of emergency responders working in the study research site of the Lower Eyre Peninsula in South Australia. TA was chosen because of its versatility and theoretical independence. The research site was selected by the researcher for different and significant reasons. These include (i) the area’s recent and severe fire history; (ii) regional people tend to appear more resourceful and self-reliant than their urban counterparts (2, 18, 21–23); (iii) the diversity of animal owners (the second of two target groups, alongside emergency responders); and (iv) geographical location—it is distant enough from large cities to require some effort and expense to visit, and hence is not “over” researched.

The reasons for engaging in a particular research topic are “never a naïve choice” (24), neither are data coded in an epistemologically free void (20). For this researcher, personal and professional interest, as a veterinarian and emergency manager, were the impetus for the choice of research topic, with the research methodology, and reflexive articulation of an ontological and epistemological position requiring thoughtful consideration to achieve the right “mix” for the project and to inform and define how best to proceed. This researcher’s situationalist orientation (i.e., that the *needs of the study* should govern a philosophical paradigm) (6) indicated a pragmatic approach as the most appropriate to enable straightforward answers to practical questions. A critical realist ontology (i.e., that knowledge might make a difference and have practical applications) and a contextualist, experiential epistemology complement this approach as realism imposes a non-pliable version of what constitutes “truth”; at the other end of the ontological continuum, a relativist or idealistic ontological position is too liberal or egalitarian for this study’s need for practical outcomes (7).

Sandelowski (25) identifies *qualitative description* as a method that provides straight answers to questions of practice and policy. *Pragmatic qualitative research* is an approach which reaches beyond plain description and ventures toward a more analytic exploration of latent meanings with more detail (6). The position of the pragmatist choosing to engage in pragmatic qualitative research is not to offer a monotone of description devoid of color, but to actively reach within these data to explore the minutiae of *prima facie* experiential detail it promises to reveal—among the “hues, tones, and textures” attributed to qualitative research *in toto* (25). Pragmatism thus “enables critique and action” (8) and was adopted in this qualitative research to achieve practical and effective solutions.

The researcher’s relationship with participants was both as an outsider, i.e., “interested observer,” and as an insider, sharing some similar training, qualifications, interests, and professional roles: a matter of which some, but not all, participants were aware (7, 26). Because of a degree of “membership” and understanding of these groups across the data corpus, a certain balance appeared to be achievable, and a conscious effort was made to be impartially, though actively, “journalistic” to strike that balance. Participants discussed emotive anecdotes about traumatic experiences and risk-taking behavior they had witnessed and were sometimes critical of animal-owner groups, thus finding the “equal and opposite” was challenging and important. At the same time, the researcher took care not to skew the interactive data collection process by seeking responses from a non-existent position. Guba and Lincoln (27) describe the researcher–participant relationship as “one of respectful negotiation, joint control, and reciprocal learning.” With this in mind, continuous re-appraisal of the research processes and evaluation of the risks of potential pitfalls helped minimize, if not neutralize, the bias of any researcher assumptions.

### Procedure—Participants and Recruitment

Some emergency responder contacts were facilitated by organizational project end users in the South Australian State capital of Adelaide. At the research site, local radio, print and television media exposure helped raise awareness of the project, as did a series of three newspaper advertisements. The researcher’s blog page contained all recruitment details and project information, including institutional ethics approval, consent forms, and contact details (28).

Two weeks prior to commencement of data collection, a site visit was made to meet with potential participants from the purposively sampled emergency responder group and to distribute information flyers in public places such as the local Council offices, public library, some retail outlets, and businesses. Representatives from all emergency services involved in first response to a bushfire in the area were specifically approached by the researcher and invited to take part: South Australia Police (SAPOL), the Country Fire Service (CFS), the Metropolitan Fire Service (MFS), and the State Emergency Service (SES).

### Procedure—Data Collection

Three focus groups and six interviews were conducted at a time and location convenient for the participants, using open-ended, semi-structured questions (Interview Guide—Primary Responders in Appendix). Not all questions were asked of all participants, and a flexible approach to the interview guide was adopted according to the context and roles of each person or group (29). Sometimes, divergent, yet, relevant topics were discussed, which fortified and enriched the data gathered. At other times, the researcher engaged in a dialog with participants, which helped to cross check meanings and draw out topics relevant to the research question.

All participants were given an information sheet and signed a consent form either prior to, or at the time of, meeting the researcher for data collection. Discussions of between 45 and 90 minutes were audio recorded, with a backup copy made by the researcher *in situ*. One copy of each data item was submitted for transcription, keeping the master files and backup copy securely on a password accessed computer and external hard disk drive, respectively.

### Procedure—Data Analysis

Thematic Analysis was chosen for analysis, because it is a flexible qualitative method not constrained by theory (7, 20). This plasticity suited the study and the researcher’s situationalist, pragmatic approach. The processes to extract detailed experiential material from these data to inform the research were largely, but not entirely inductive, and largely, but not entirely contextualist. The analysis, therefore, moves from descriptive to interpretative when meanings are sought—and needed—to extract answers to the particular question posed of this *data set*.

In data driven, inductive TA, coding is undertaken without the constraints of pre-existing categories. That being said, the overall research question applied to the data corpus contains elements for which the researcher was keenly watching while coding the data set.

Once transcribed, the researcher cross checked the printed transcripts for accuracy by playing back the audio files, and making corrections, both on the hard copy and the electronic version. Before coding began, “data familiarization” took place, by again reading each data item carefully three or four times.

The recursive process of analysis and data driven coding yielded 155 codes. Data were managed using the CAQDAS^1^ system, NVivo 11, and on a parallel Excel spreadsheet. The reason for using the supplementary spreadsheet was primarily to enable the researcher to “look” at these data from a different perspective and also provide a form of visual thematic map. The spreadsheet was used up to, and including, the development of themes and subthemes but did not extend into data extraction.

Next, codes on the spreadsheet were grouped into clusters of “like” codes. It was interesting to note that when considering patterns across the data set, these clusters did not translate in their entirety into themes. The final 10 themes actively identified by the researcher comprised codes from different groups as the central organizing concept of each theme was distilled. A thematic map and table (Table 1) (other than the tabular form of the spreadsheet) was generated to visualize and enhance the interrelationships and logical structure of the themes and subthemes of data analysis. This paper discusses the first of those themes.

## Interpretative Analysis and Discussion: Bushfire in Kingdom Animalia

Bushfires can affect all taxonomic Kingdoms. This paper’s focus is upon members of *Kingdom Animalia*, specifically, human beings and the non-human animals they own or enjoy. Just as taxonomy is in a permanent state of flux and revision, so too are the rules, recommendations, and management tools associated with bushfire emergencies. A serious fire-affecting people, their livelihoods and microclimates, is a complex *non-routine social problem* (30). The discernment of how people and emergency managers can better equip communities to protect themselves, and the things they hold dear, including their animals, is an indisputable imperative given the evidence-based predicted changes to near-future global weather events.

### Theme 1: Bushfire and Animals Shouldn’t Mix

Animals on a fireground compound the challenges and complexity this natural hazard presents to their owners, emergency responders, and others in the community. They commonly invoke a variety of human reactions and responses, some of which are very unsafe, and some representing a close encounter with mortality. Many fall into the category of “good luck rather than good management,” and others—considerably less than fire prevention authorities would like—represent the outcome of thoughtful and practiced planning. Within this range is found every conceivable permutation of response behavior as unique as the people comprising them. The animal is like the pebble in the pond, and the emanating ripples represent the diverse and potentially far-reaching human consequences of animals being part of the preparedness and response equation.

The ideal situation where animals are absent from a fireground is extremely unlikely. The opposite end of the spectrum, where the presence of animals may contribute to a chain of events which can lead to tragedy and human death, is more probable, with incidents involving animals identified as a reason why people take risks (31, 32). Nobody likes to think of animals being burned to death, and as one interviewee said: *losing horses in a fire* …*. is one of the worst things in the world to see. It’s terrible*. However, animal welfare cannot be viewed in isolation—because it is not a “stand alone” issue in enacting an effective emergency response.

Shane, a senior fire-fighter, detailed how people’s emotions can supersede self-preservation.

We talk about the emotion that’s attached to children and families. I think you can almost double it for dogs, cats, and horses. And horses, in particular, seem to attract a hell of a lot more emotion from the people who are attached to them. The amount of grief that a horse owner can cause themselves in their attempts, vain attempts nine times out of 10, to get to their horses is incredible.

Shane went on to describe a situation where some of his crew disregarded orders and went to rescue a horse from a burning stable. If even trained firefighters make emotional decisions because of animals—which they probably did not own—it is not surprising that animal owners also adopt unsafe behavior. Every animal cannot be saved from a fire, but current warnings do not necessarily resonate enough to overrule basic and innate human drivers to attempt the rescue of dependent others (31, 33).

The subsequent psychological trauma and reliving of a distressing fire event may be overcome, or it could linger for a lifetime (34–37). Of the four firefighters in Shane’s example above: “… *one rang his wife to say goodbye, literally* … *Two of them received counselling for three years, severe counselling, like, they needed it*.” Similarly, a farmer evacuating horses while a boarding kennel on the adjacent property, full of cats and dogs, describes hearing the animals’ cries as the buildings burned and says he will never stop hearing that sound in his head.

People also take risks when they return to a dangerous place prematurely to retrieve or move animals, or when a family’s departure is delayed by attempts to catch animals they want to evacuate with them. Just such a scenario was related by Jayne, an experienced fire officer with 10 years experience in a rural, at-risk community, and who works in the area of fire safety and community outreach:

You must include your animals (in your bushfire plan) because—imagine a family with kids and if mum’s only focus is “Let’s get the kids in the car. Let’s get the kids in the car.” But the kids are focused on the cat and the dog … if the cat scarpers and the dog hides under the shearing shed, then you’ve got kids running off after animals and the mum’s trying to run off after the kids and—it just adds all that unnecessary worry and stress and anxiety.

Shane also related some unsafe practices he has seen animal owners adopt.

We’ve had instances where people have released horses on the roads and it has been nothing short of a miracle that we haven’t worn one in the truck or a member of the public hit them. They should never be put on the road, in my opinion. By all means open every internal gate. It allows them the freedom of movement and again, it’s acceptance by the community in the area we live in.

Releasing animals onto public roads results in a different, but equal risk, to their safety, threatens public safety, and can leave people with no escape route. A collision between a motor vehicle and a large animal loose on a road is very likely to injure people in the vehicle, as well as the animal. Ambulances may or may not be able to access the location. Responders are then faced with possible entrapment of people in the vehicle, which could be a fire truck. The diverted or immobilized crew could be placed in life-threatening danger, or, be thwarted in their mission to assist someone else. People are also likely to be distressed by a severely injured animal they are unlikely to have the time, resources or training to be able to help. A catastrophic outcome is preceded by a cascade of component negative events often stemming from one avoidable act, error, or omission. It is the underlying decision-making that needs scrutiny (Westcott 2015, unpublished data).

Focus group member, Kate, offered the counterpoint: “*the theory behind opening up all the gates was that animals will find their own way to a safe spot*.” Kate explained this has been a relatively common practice on broadacre farms in the past. But what may be appropriate in a sparsely populated broadacre farming community does not translate into a relatively densely settled peri-urban population and landscape. Potentially adverse consequences are overlooked as the focus is on the animal, and their owners can, quite subconsciously, invert the well-known and legislated hierarchy of protection, *life, property, and environment* (38). Development on the urban fringe, often with allotments large enough for small-scale animal keeping, can place residents with the least fire experience in a vulnerable position, risking decision-making based on folklore or myth (39, 40).

The safety of the animal *is* the owner’s responsibility, as is the ultimate fate of that animal, although there are occasions where nothing could save a catastrophic situation. The phenomenal speed of a crop fire is an example, but so too is the presence of underlying pathology in an animal, diagnosed or otherwise, which can be fatally exacerbated by the stress of a fire, and peri-fire events.

If animals are lost and people are ill-prepared, they may experience feelings of guilt, or might rationalize the situation to themselves by believing they have done “the best they could.” For example, releasing the animal onto a road could be viewed as “giving the animal a chance,” and give the owner a feeling of having “done something”—bringing some comfort to the owner, who is then free from having to manage that animal, and can focus on other things. This is not likely to be the motivation for releasing animals, but could creep into a person’s consciousness given an immediate or impending threat where preparations, for whatever reason, are less than optimal.

Shane highlighted two extremes:

If we’ve got people that are gonna’ stay and defend and leave their horses out in the paddock, they’re kidding themselves. If they’ve decided that their action plan on this day with a total fire ban … their horses can come up to the stables. If they’re serious about it, they’d put sprinklers in or they do whatever it is to make a fuel reduced zone to try and ensure the survival of those animals. That’s a plan. (Horses) sitting in the bottom paddock when the husband’s out fighting the fire and the missus can run down on the four-wheeler and get them, that’s not a plan. That’s the start of a fatality. And I will point that out very bluntly—politely—I ask them to leave their dental records on the table in a fire proof sheet.

Responders certainly notice when animal owners are organized and act safely and with forethought. Amid the inevitable chaos, this glimmer of order stands out like a beacon. It can be a very simple and easily managed response. Zoe in one of the responder focus groups commented: “*people with cats and dogs, they will stick them in cars and they’ll just go. They tend to grab their family pets pretty quick*.”

Barry, an operational firefighter in another focus group, observed:

Some people are fairly or ganized—there was a fire at (town name) and I went down the front street and there were people with horse floats^2^ (trailers) down there and it was a very visible fire so people were panicking in town, I remember seeing a lot of horse floats down the front street. So some people are organized.

At another fire, Jack noticed:

The interesting factor was that a lot of the people who were living around there went to a lot of work to remove the horses to try and get them out. They got a lot out, and they did a very, very good job on a voluntary basis. A lot of people just came and said, “I’m here. Here’s my horse float, put it in there, and we’ll put it somewhere.” I found that really heartening.

To Barry and Jack this seemed “organized,” but moving animals when the fire is “very visible” represents considerable risk. Undeniably, the logistics of moving horses multiple times during a summer can be substantial—inconvenient, costly, and time consuming.

Ben, in one of the firefighter focus groups, said:

If you got an animal and you wanna’ look after it, you plan for it, and know how long it’s gonna’ take to get that horse on the float. And if there’s no fire, there’s no fire. You don’t get a great deal of catastrophic days or extreme fire days through a year, but you might have to move them—animals—10 times, and it might be the tenth time that there’s a fire. But we have a lot of days that are really hot and all that, and people get complacent.

Jayne identified reasons why farmers are a group of animal owners who take preparedness seriously, for reasons of economics and animal husbandry: “*Most stock owners* … *prepare because it’s their livelihood, their income, their business continuity. And that might include generations of breeding. Most land owners and stock owners are reasonably well prepared if not very well prepared*.”

Other companion animal owners she knows also make good plans:

I know of one person … on catastrophic days, her workplace is closed, so she’s home. But on severe and extreme days, she has a permanent booking at the local boarding kennels, because she doesn’t have family in town.

There’s someone else with a pet python. So she pops him in two calico bags and takes him very discreetly in a bag and pops him at her feet at work. No one would know what’s in the bag … he’s restrained and it works. She brings her dogs into town to relatives and doesn’t have to worry, or waste time, or try to rush home or anything like that.

Shane positively recalled an example of timely collaboration among one group:

The local pony club are very, very good. They actually bring their horses into clean areas like fuel reduced zones. There are people there with firefighting gear and that’s what they do. They start to talk about it on catastrophic days and they use their social networks to bring the horses in and reduce the amount of movement that’s required.

Barry mentioned that relocating and planning for moving animals was not given enough emphasis.

If it was pushed a lot harder … if there is gonna’ be a catastrophic day, you pull your horses out maybe the day before. When I moved out of home, my animals used to go with mum and dad in town that day or that morning, if there was gonna’ be a catastrophic warning, but I was in that position where I was able to take my animals somewhere. I think if there was more education on getting the animal out a day before, even if there’s no fire and it might cost you a few dollars to do that, but you’re not gonna’ stuff around on that day when there’s a fire and get in everyone’s way to try and move animals—maybe move them a day before.

Traffic congestion seems to be a frequently overlooked problem that many animal owners fail to consider. Shane commented that:

There are still others that think, “Oh, look there’s a fire. I’ll grab the horse float and go rescue my horse.” Add to the traffic and add to the congestion. The float’s not hooked up, they don’t have an escape plan, they don’t have a horse that they can get in a horse float without 10 people. All the vagaries of dealing with a big excited animal … rarely get taken into account, neither the reality of the circumstances and the surrounding fuel loads. What are your horses like in smoke and that north wind environment?

These are problems which should be straightforward to solve. The relationship between animal owners and emergency services should be one of partnership and will be enhanced and mutually beneficial if just a little proactive preparedness and reciprocal consideration is exercised. Owners need to recognize that traffic congestion can become a major problem for safe and effective movement of emergency vehicles and for public traffic moving to relief centers or other designated safe areas during an emergency incident. Considerate arrangements to be clear of potential traffic bottlenecks ahead of likely peak use will be greatly appreciated by emergency responders. Animal husbandry and animal behavior may be something which new owners need help to manage and some collaborative mentoring of novice owners could be useful. Stock and horse trailers need regular maintenance and may or may not be in frequent use. No plan is perfect, but better outcomes will be achieved when a plan exists, particularly, if it is enacted early. Jack commented: “*It’s always gonna’ be crazy. It’s always gonna’ be chaos. Nothing—any plan that you’ve got is only gonna’ last until it faces reality*.”

In Australia, once residents and other non-responders leave a fireground, they are not permitted to return until it is declared safe to do so—which could be days (41). People evacuating multiple animals sometimes arrange a “shuttle” of transport, where animals are unloaded at a roadblock and either reloaded or walked out, so that the person “on the inside” can legally ferry animals up to the road block.

Zoe has often been assigned to road blocks:

And lots of people have got, like, four horses, but they’ve only got a two-horse float. If they get that float out for the first two horses, they can’t get it back in. What do they do? So they’ll walk them out. They’ll ask where to go and we’d say, “Look, sorry, we don’t know, but you need to clear the road.” So we’re more concerned with getting them out of the way. They were leading horses out … where the hell do we send them?

Transporting animals before roadblocks are in place can also be problematic. Jayne talked about how moving animals in a more timely fashion is still challenging: *if you’ve got a single horse float and two horses, that means you’ve got to make two trips. Have you thought about that?*

Interviewees Jack and Joe, both in the police focus group, suggested another reason:

People don’t like leaving the security of their home—disrupting their home life. Just that people seem to be reluctant, I think, to move the animals even though they know the fire is coming. And they seem to leave it all to the very last minute. And then they’re finally rounding up horses and all that sort of thing and you think—I don’t know why you didn’t do it five hours ago.

The perceptions of the participants who contributed to this data set, all of whom are experienced first responders, tell us about the way animal owners behave. Have they observed a key, common denominator, and/or particular difficulties for animal owners? One of the focus groups talked extensively about two aspects they felt needed attention—allocating safe areas where animals can be taken when people evacuate or relocate on catastrophic days and for owners to be more timely in activating their bushfire survival plan.

The first of these is a perennial topic for discussion because on face value, a solution sounds easy—but in reality is fraught with difficulty. Even in the country, dozens of vehicles all together converging on a central “safe” haven for animals will cause traffic chaos, blocking the paths of emergency vehicles and possibly increasing the incidence of motor vehicle accidents. In the city, with proportionally larger numbers of vehicles approaching a common destination, more problems could be created than solved. Holly, in the group said,

Somebody needs to bite the bullet and designate one of the ovals (for animals). Horses—if they can get to the racecourse is probably best—you can’t have horses running around the oval with kids. And the people running the ovals don’t want horses churning up the turf and leaving manure everywhere. So it’s feeding, and cleaning, and also who’s gonna be responsible if there’s any damage, or injury to people? So until we overcome those sorts of problems … it’s an insurance nightmare. And at the school, they didn’t want to have animals in there again because it made massive marks on the gym floor that had to be repaired, but what’s the alternative?

Having dogs and cats, rabbits … in the same evacuation area as people and children, hopefully, they’re in pet packs or something like that, but it’s still massively distressing for them. It’s not fair on the animals. It’s not fair on the people.

However, the social responsibility remains, and a solution needs to be found for unacceptable situations such as the older lady who spent most of the (catastrophic) day in her car in the supermarket car park with her cat, dog, and two chooks.

As global temperatures rise, severe bushfires in Australia and elsewhere are the “new normal” (Campbell, D. South Australian CFS, personal communication, 2016), and prevention is vastly less costly than response and recovery (42). Converting an intention (to do something not enjoyable) into a routine action (as non-threatening and essential as buying groceries) could mean a substantial shift in the uptake of readiness behavior.

## Conclusion

Analysis of these data in this paper supports the premise that reciprocal collaboration between emergency services and animal owners can positively contribute to the efficient progress of a response, with mutual benefit and an enhancement in positive outcome expectancy. This includes more effectively implementing practical solutions to important potential problems such as traffic congestion. Reducing the number of non-essential vehicular movements at the time of an incident significantly improves the ability of emergency services to readily access affected areas. Thus, as these data show, moving animals in anticipation ahead of a day of catastrophic fire danger has the double advantage of reducing traffic at critical times, and removing animals from a high risk area, with the added benefit of allowing their owners to make safe arrangements for themselves.

Designating safe places to take animals remains problematic despite ongoing discussion among response and recovery agencies. Animals are the responsibility of their owners, as is their safety, and/or relocation to a place of refuge. However, special needs groups of people who require help to do this, or who negotiate their social microclimate in the company of an assistance animal, need support and a solution to this potentially distressing and possibly life-threatening problem.

In addition to identifying reciprocal collaboration as mutually beneficial, this analysis identified two key outcomes (i) gaining an understanding of the etiology of behavior and decision-making and (ii) offering practical suggestions to influence policy development and implementation. These are summarized in Table 2, and are the subject of later data analysis.

**Table 2:** **Key outcome themes identified in the current study**.

Policy development and implementation	Etiology of decision-making
Catastrophic day leave	Effect of the social microclimate
Financial incentives	Adaptive rewards as opposed to costs—achieving a net gain
Insurance policies Municipal fees and charges Best practice rewards	
	Maladaptive costs—negate maladaptive rewards
Farming practices, fuels, and firebreaks	Dynamic risk assessment

Future research, including analysis of the remaining themes of this data set, will further explore and address these issues from different perspectives, and continue to assess how the interface between animal owners and emergency responders can improve the safety and survival of animal owners, and of other groups. Procedures and processes, strategies, and tactics that are of assistance to animal owners are likely to be translatable and applicable to other areas of need where gaps exist. Additionally, some bespoke solutions may be needed, and could be formulated, trialed, and expanded as required to enhance bushfire survival.

Further research will then be needed to evaluate the efficacy of policy changes suggested by this study overall and to ascertain the role and application of relevant social theory in maintaining and enhancing community well-being, and, ultimately, the saving of human life in a bushfire emergency.

## Ethics Statement

This study was carried out in accordance with the recommendations of the National Statement on Ethical Conduct in Human Research (2007), and the Human Research Ethics Committee of Western Sydney University, approval number H11118, with written informed consent from all subjects. All subjects gave written informed consent in accordance with the Declaration of Helsinki. The protocol was approved by the Human Research Ethics Committee of Western Sydney University.

## Author Notes

“Don’t Just Do Something … Stand There!” is a quotation used in training work-shops by Robert Kearney, Order of Australia Medal, Justice of the Peace, Vietnam veteran, military historian, author and retired trainer to the South Australian Country Fire Service.

## Author Contributions

RW and MT contributed to the initial design of the research project on which this manuscript is based, and all authors contributed to refinement of the design and research that is currently underway in support of this. RW drafted the manuscript and MT, KR, and HB contributed to revisions. All authors read and approved the final manuscript.

## Conflict of Interest Statement

The authors declare that the research was conducted in the absence of any commercial or financial relationships that could be construed as a potential conflict of interest.

## Footnotes

^1^Computer-Assisted Qualitative Data Analysis Software.

^2^The Australian term, “horse float” is equivalent to the North American “horse trailer.”

## Appendix

### Interview Guide—Primary Responders

1. Background: hazard severity
  - How does “fire season” seem to have changed over the last 10–15 years?
  - How does a response begin?
    ○ Resources available and/or deployed
    ○ “Situation reports” and communications
    ○ Decision-making—local knowledge, prior experience, precautionary actions, intelligence from first-on-site, and influence of ambient conditions
    ○ Is it assumed that a fire could reach (the highest) Level 3
  - Likelihood of occurrence—how are prevention and preparedness messages communicated in off-season? Are particular demographics targeted?
  - As a community member, how does being trained as a firefighter/emergency services member change attitudes to a bushfire hazard?
  - Are firefighters/first responders as individuals, each a conduit of information out into the community?
  - How do firefighters/responders deal with being away on a fire truck, when their own homes or properties may be under bushfire threat?
  - Hypothetically, if a fire crew goes to the assistance of a household who have stayed to defend, but for whatever reason that is no longer tenable, and the crew and the residents are in danger as a single group—the fire crew would take control of the situation—how collaborative can such an action be between both parties, or can it?
  - How do the fire crew enforce “life, property, and environment” in circumstances when people may be attempting to gather possessions, or save animals?
2. With respect to animals
  - What kinds of animal issues have arisen during a bushfire response?
    ○ Companion animals
    ○ Assistance animals
    ○ Horses
    ○ Livestock
    ○ Wildlife
    ○ Animals wandering at large
    ○ Businesses, e.g., boarding kennels
  - Getting back to *life, property, and environment*: how do you manage the “gray” areas, such as livestock as property
  - Have fire crews picked up animals in field, or are they given animals (especially wildlife) by members of the public?
  - What do they do with them?
  - Does this detract from core business?
  - Fire crews can be distressed by issues of animal welfare during a fire—how do you manage this?
    ○ Debrief/after action review
    ○ Can it detract/distract from response/team efficacy?
  - What about home owners—wanting to return home to attend to livestock/pets/animals in their care?
  - How do primary responders perceive animal owners, with respect to expectations of behavior in a fire, and their capabilities?
  - Have animal owners caused difficulties for responders in-field? e.g., with well-meaning people (with disregard for their own safety) attempting to rescue animals from a fire? Are these people animal owners? Others unauthorized? Media?
  - What interaction or support has there been locally with existing animal agencies—Primary Industries and Resources SA (PIRSA), Royal Society for the Prevention of Cruelty to Animals (RSPCA), Department of Environment, Water and Natural Resources (DEWNR), Council or Shire Ranger etc?
  - What are your observations of animal owners in the field? e.g., levels of preparation, eagerness to cooperate?
  - Are there challenges for responders with respect to assistance they may be called upon to give, or wish to give, animal owners or to animals directly; or with respect to what they should do, what they can do, and what they actually do? (behavior in the field vs. policy)
3. Information gathering and organizational cohesion/communication
  - How is information sourced in a response, as an input (e.g., field intelligence) and an output (information and advocacy back to community)?
  - Within the organization, how is local knowledge integrated with centralized directives?
  - How is information given to the community assessed for accuracy and timeliness?
  - How is accuracy balanced with giving the community information early in the event timeline?
  - Is organizational credibility (e.g., public perception of the “brand”) consciously used to engage community?
  - Have problems with animals and/or their owners presenting in-field adversely affected community relations?
  - Has this been addressed in After Action Reviews?
  - How well does interorganizational cooperation/collaboration work—primary responders with PIRSA, RSPCA, DEWNR, Council Ranger, etc?
  - Individual and community knowledge base—are there particular groups or demographics which are difficult to engage?
  - Are there groups which could benefit from bespoke response options?
  - What training courses or workshops are available for the public, and do these include identified special groups, such as animal owners?
